# Integrated Analysis of lncRNA and mRNA Reveals Novel Insights into Wool Bending in Zhongwei Goat

**DOI:** 10.3390/ani11113326

**Published:** 2021-11-22

**Authors:** Xiaobo Li, Zhanfa Liu, Shaohui Ye, Yue Liu, Qian Chen, Weijun Guan, Yabin Pu, Lin Jiang, Xiaohong He, Yuehui Ma, Qianjun Zhao

**Affiliations:** 1 Institute of Animal Science, Chinese Academy of Agricultural Sciences (CAAS), Beijing 100193, China; libobo2922@hotmail.com (X.L.); liuyue196160@163.com (Y.L.); chenqian602141@163.com (Q.C.); guanweijun@caas.cn (W.G.); puyabin@caas.cn (Y.P.); jianglin@caas.cn (L.J.); hexiaohong@caas.cn (X.H.); 2 Department of Animal Breeding and Reproduction, College of Animal Science and Technology, Yunnan Agricultural University, Kunming 650201, China; 3 Ningxia Hui Autonomous Region Breeding Ground of Zhongwei Goat, Zhongwei 755000, China; xqycbj@126.com (Z.L.); ysh@ynau.edu.cn (S.Y.); 4 CAAS-ILRI Joint Laboratory on Livestock and Forage Genetic Resources, Institute of Animal Science, Chinese Academy of Agricultural Sciences, Beijing 100193, China

**Keywords:** goat, long non-coding RNAs, wool bending, hair follicle, mRNA

## Abstract

**Simple Summary:**

The high-quality lambskin of the Chinese Zhongwei goat has a high economic value. The quality of lamb skin is mainly affected by the curvature of the wool, which is regulated by the growth and development of hair follicles. In this study, the expression profiles of long non-coding RNAs (lncRNAs) of 45-day-old and 108-day-old Zhongwei goats were constructed by the Ribo Zero RNA sequencing. A total of 60 differential lncRNAs and 352 differential mRNAs were identified. The functional annotation of differential lncRNAs target genes showed that they were mainly enriched in PI3K-Akt and Arachidonic acid metabolic signaling pathways. In combination with qRT-PCR and WGCNA results, we speculate that *LOC102172600* and *LOC102191729* might affect hair follicle development and wool curvature by regulating the target genes. These results provide new insights into the potential role of lncRNA in regulating wool bending.

**Abstract:**

Chinese Zhongwei goat is a rare and precious fur breed as its lamb fur is a well-known fur product. Wool bending of lamb fur of the Zhongwei goat is its most striking feature. However, the curvature of the wool decreases gradually with growth, which significantly affects its quality and economic value. The mechanism regulating the phenotypic changes of hair bending is still unclear. In the present study, the skin tissues of Zhongwei goats at 45 days (curving wool) and 108 days (slight-curving wool) after birth were taken as the research objects, and the expression profiling of long non-coding RNAs (lncRNAs) and mRNAs were analyzed based on the Ribo Zero RNA sequencing (RNA-seq) method. In total, 46,013 mRNAs and 13,549 lncRNAs were identified, of which 352 were differentially expressed mRNAs and 60 were. lncRNAs. Functional enrichment analysis of the target genes of lncRNAs were mainly enriched in PI3K-Akt, Arachidonic acid metabolic, cAMP, Wnt, and other signaling pathways. The qRT-PCR results of eight selected lncRNAs and target genes were consistent with the sequencing result, which indicated our data were reliable. Through the analysis of the weighted gene co-expression network, 13 co-expression modules were identified. The turquoise module contained a large number of differential expressed lncRNAs, which were mainly enriched in the PI3K-Akt signaling pathway and cAMP signaling pathway. The predicted *LOC102172600* and *LOC102191729* might affect the development of hair follicles and the curvature of wool by regulating the target genes. Our study provides novel insights into the potential roles of lncRNAs in the regulation of wool bending. In addition, the study offers a theoretical basis for further study of goat wool growth, so as to be a guidance and reference for breeding and improvement in the future.

## 1. Introduction

The phenotype of wool depends on the growth and development of the hair follicles. A previous study showed that lncRNAs participate in gene expression regulation and protein synthesis during hair follicle development [1]. The Zhongwei goat is a unique native breed producing white fur in China. The fur obtained from one-month-old lamb has a flower pattern with a wool length of 7 cm. The wool bending feature will disappear in the process of growth. Its economic value and quality will diminish with the gradual disappearance of the curls. In this paper, the Zhongwei goat was taken as the research object so as to explore the regulation mechanisms of lncRNAs on the phenotypic changes of wool bending. Although several studies on wool phenotypes have been performed [2,3], the mechanisms underlying the change of Zhongwei goat wool curves is elusive and needs to be explored.

Long non-coding RNAs are a class of RNA whose length is larger than 200 bp without encoding functional proteins, which play a key role in regulating the expression of protein-coding genes [4,5]. LncRNAs, previously considered to be part of transcriptional noise, are now a new regulatory layer for transcriptional and post-transcriptional gene regulation [6,7]. LncRNAs exert their function by binding or interacting with proteins, RNA, and DNA [8]. In recent years, some studies have been performed to identify lncRNAs affecting the growth and development of hair follicles, as well as wool growth. For example, the non-coding RNA expression profiles of Hu lamb skin were constructed, and *TCNS_00279168* was predicted to regulate the target genes *FGF12* and *ATP1B4* [2]. A series of lncRNAs, including *ENSCHIT00000009853*, *MSTRG.16794.17* and *MSTRG.17532.2*, were shown to be potentially associated with cashmere fineness through transcriptomics analysis of the skin tissues of Tibetan cashmere goats [9]. The expression profiles of lncRNAs and mRNAs of the skin from Hu sheep with large, medium, and small waves in the wool were constructed, and the differentially expressed lncRNAs were determined [10].

Wool crimp is caused through the regulation of a large number of genes on the hair follicles. Some studies have reported that the asymmetric division of hair follicle cells contributes to hair bending [11]. The differential pressure exerted by asymmetric cell division and cell hardening on the bottom layer of the hair follicle causes fiber bending, and the degree of bending is regulated by keratinization [12]. The hair follicle is the miniorgan that produces hair and is critical to determine the growth of wool [13,14]. Therefore, it is of great significance to determine the key genes, signal transduction pathways, and regulatory mechanism of hair follicle morphogenesis. It has been found that lnc-000133 and lncRNA-HOTAIR are involved in the growth of goat hair follicles [4,15]. LncRNA *MSTRG.223165* was identified to participate in wool follicle development by acting as an miR-21 sponge through the transcriptomic analysis of Chinese Aohan fine-wool sheep [16]. Through an integrated analysis of the lncRNAs and mRNAs expression profiling of the skin samples from fetal and lamb Subo Merino, Ablat Sulayman et al. identified 471 differential lncRNAs and some key pathways such Wnt, TNF, and MAPK signaling pathways related to the growth and development of hair follicles [1].

Although some progress has been made in regulating hair phenotypic changes, as well as for the growth and development of hair follicles, there are few reports on lncRNAs related to hair bending changes. The molecular mechanism underlying the change of the wool bending phenotype in the Zhongwei goat is not clear. The present study is aimed to construct expression profiles of lncRNAs and mRNAs of Zhongwei goat skin with different wool bending phenotypes, and to identify the differentially expressed lncRNAs involved in the biological processes of hair follicle cell growth and development using the Ribo Zero RNA sequencing method. This study provides new insights into the molecular mechanisms underlying goat wool bending.

## 2. Materials and Methods

### 2.1. Experimental Animals and Sample Collection

The Zhongwei goats used in this experiment were provided by the Zhongwei goat breeding farm in Zhongwei City, Ningxia Hui Autonomous region, China. The feeding conditions of these goats remained the same throughout the period. Three lambs were randomly selected for in vivo sampling, and the shoulder skin tissues of 45-day-old (curving wool) and 108-day-old (slight-curving wool) lambs were collected. As the same individual was sampled twice in different periods, the sampling position needed to be axisymmetric. Systemic anti-infection treatment was performed after sampling.

### 2.2. RNA Extraction and Sequencing

The RNA was extracted from the samples stored in RNAlater reagent for sequencing (RNA-seq). The total RNA of six samples (each three individuals at 45 days old and three individuals at 108 days old) was extracted using RNeasy Plus Universal Mini Kit (QIAGEN, Dusseldorf, Germany) according to the instructions on the kit. The purity of the RNA was preliminarily quantitatively determined by a NanoDrop 2000 spectrophotometer (Thermo Scientific, Wilmington, DE, USA), and the concentration of RNA sample was accurately quantified by Aglient 2100 (Agilent Technologies, Santa Clara, CA, USA). Standard-compliant RNA (RNA total amount > 2 μg, concentration ≥ 100 ng/uL, RIN ≥ 7, OD260/OD280 = 1.8–2.2, OD260/OD230 = 1.8–2.2) was used for the library construction.

Ribo-Zero removal kit (Epicentre, Charlotte, NC, USA) was used to remove the rRNA from the total RNA sample (some lncRNAs have the same polyA tail structure as mRNAs, and the method of removing rRNA can maximize the retention of lncRNAs with a polyA tail). Through the Illumina Hiseq 2500 platform of the Berry NGS Company (Beijing, China), the library was constructed. The reads containing adaptor, N more than 10% of reads, and low-quality reads were removed from the raw data, and quantity control of clean data were performed by calculating Q20, Q30, and GC contents. The clean data were aligned with the reference genome using the tophat2 software [17]. TopHat2 was applied to identify the splicing sites between the exons. The transcripts of a single sample were assembled by Cufflinks (v2.2.1) [18], and the results were integrated by comparing with the reference genome by Cuffmerge. Finally, the assembly result of the merged transcript was compared with the known transcript using Cuffcompare. Transcripts with a length ≥ 200 bp, exon number ≥ 2, and minimum coverage ≥ 3 were further analyzed using the following four tools, CPC (Coding Potential Calculator) [19], CNCI (Coding-Non-Coding Index) [20], PLEK (predictor of long non-coding RNAs and messenger RNAs based on an improved k-mer scheme) [21], and Pfam (the protein family database) [22].

### 2.3. Prediction of lncRNAs Target Genes

LncRNAs mainly regulate the coding region of target genes through cis-acting or trans-acting. Cis target genes of lncRNAs were predicted using the software LncTar [23]. The WGCNA R package (v1.68) was used to analyze the correlation or co-expression of lncRNAs and protein coding genes among samples to predict the trans target genes [24].

### 2.4. Weighted Gene Co-Expression Network Construction (WGCNA)

The lncRNAs expression data were analyzed with WGCNA R package (v1.68). We constructed the weighted gene co-expression network of all the differentially expressed lncRNAs and divided them into different modules according to the expression pattern. The correlation between different modules was analyzed, and all modules were clustered.

### 2.5. qRT-PCR Analysis

**Figure 1:**
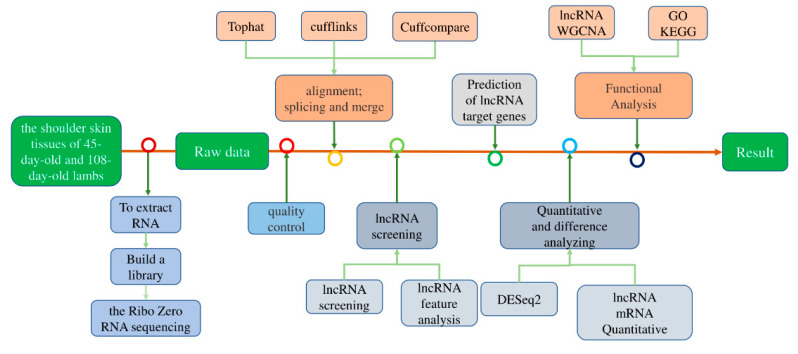
Work flow chart of sequencing data processing.

The total RNA of six skin samples was extracted and each of them was transcribed into cDNA three times for quantitative detection. The reaction system of 20 µL prepared with TB Green^®^ Premix Ex Taq resin II and cDNA was quantified in a QuantStudio^TM^ 7 Flex 96-well System (Thermo Fisher Scientific, Waltham, MA, USA). The reaction temperature was held at 95 °C for 30 s, followed by PCR stage of 95 °C for 5 s, and 60 °C for 34 s, and the melt curve was at 95 °C for 15 s, 60 °C for 1 min, 95 °C for 15 s. Forty cycles were carried out under this condition. The data were analyzed using the 2^−ΔCT^ method. The goat GAPDH gene was used as the reference gene to normalize the target gene data. For the above experiments, we carried out a workflow flow chart (Figure 1).

### 2.6. Differential Expression Analysis of lncRNAs and mRNAs

The differentially expressed genes between group D45 (curving wool) and D108 (slight-curving wool) were analyzed using DESeq2 [25]. |log_2_FC| ≥ 1 and *p*-value < 0.05 were used as the screening criteria to detect differentially expressed genes. Functional annotation and enrichment analysis of cis-acting and trans-acting differentially expressed lncRNAs target genes were performed by GO and KEGG. Enrichment analysis of differentially expressed gene sets was done using GOseq R [26] and KOBAS 3.0 [27] software packages. GO terms and pathways with *p*-value < 0.05 were defined as significant enrichment in the differentially expressed genes.

## 3. Results

### 3.1. Whole Transcriptome Sequencing Results

To systematically investigate the identification and characterization of Zhongwei goat’s wool growth, we performed cDNA sequencing on six skin samples at the two postnatal stages (day 45 and day 108) of the Zhongwei goat. After removing the low-quality reads and adapter fragments, we obtained 332,078,170 reads in all of the libraries, which corresponded to 93.35% of the raw reads. The number of clean reads in the group for day 108 were 48,700,561, 60,520,557, and 63,988,208 (Table 1), with GC content of 46.05%, 45.11% and 46.45%, respectively. The number of clean reads in the group for day 45 were 53,480,008, 52,662,688, and 52,726,148, with a GC content of 47.09%, 46.22% and 45.44%, respectively. The Q20% in all the libraries were above 97%, indicating that the clean reads were of a good quality; 94.39–95.15% of the clean reads in all the libraries were successfully mapped to the reference genome of Capra Hircus (CHIR 1.0).

### 3.2. Identification of lncRNAs and mRNAs in Zhongwei Goat Skin

**Figure 2:**
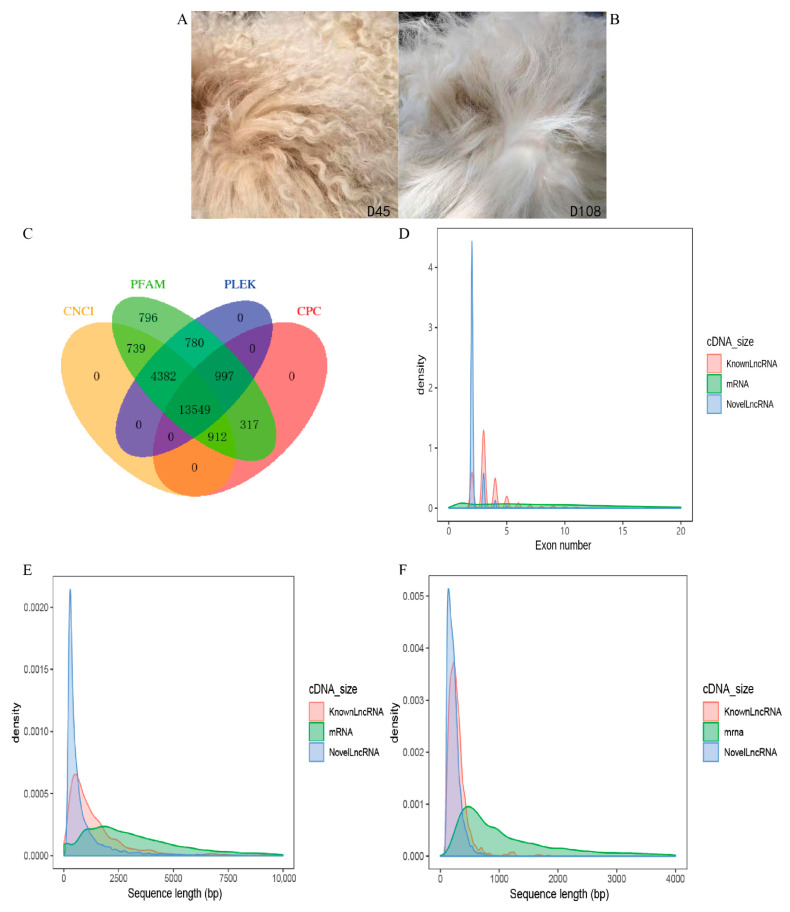
Identification of lncRNAs and comparative characteristic analysis between lncRNAs and mRNAs: (**A**) the wool of a 45-day-old Zhongwei goat; (**B**) the wool of a 108-day-old Zhongwei goat; (**C**) Venn diagram of the lncRNAs identified by four coding potential screening tools (CPC, CNCI, PLEK, and FPKM) and the intersection part was selected as the candidate lncRNAs for follow-up analysis; (**D**) the exons number of mRNAs and lncRNAs; (**E**) the length distribution of mRNAs and lncRNAs; (**F**) the open reading frame (ORF) length of mRNAs and lncRNAs.

**Figure 3:**
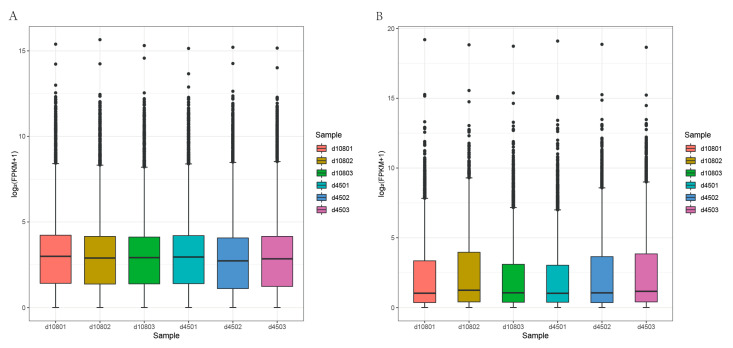
The expression of mRNAs and lncRNAs in six samples at two developmental stages: (**A**) mRNAs expression; (**B**) lncRNAs expression.

A total of 46,013 mRNAs and 13,549 lncRNAs were identified by four mainstream coding potential analysis software (CPC, CNCI, Pfam and PLEK) (Figure 2C). Comparing the obtained lncRNAs with mRNAs, the length of lncRNAs range was mainly between 200–1000 bp, the lncRNAs included 2–4 exons, and the mRNAs consisted of 1–20 exons (Figure 2D,E). The ORF sequence of mRNAs was extracted by annotating the known gene structure, and the ORF sequence of lncRNAs was predicted by ESTScan. The obtained ORF sequences were transformed into protein sequences for statistical comparison. Most of the ORF length of lncRNAs was between 100–1000 bp, and most of the ORF length of mRNAs was between 100–2000 bp (Figure 2F). The comparison of the lncRNAs and mRNAs expression levels was estimated by FPKM (fragments per kilo-base of exon per million fragments mapped) using Cufflinks software components. The results showed that the expression levels of lncRNAs transcripts were relatively lower than that of the mRNAs (Figure 3).

### 3.3. Differential mRNAs and lncRNAs Expression Levels

**Figure 4:**
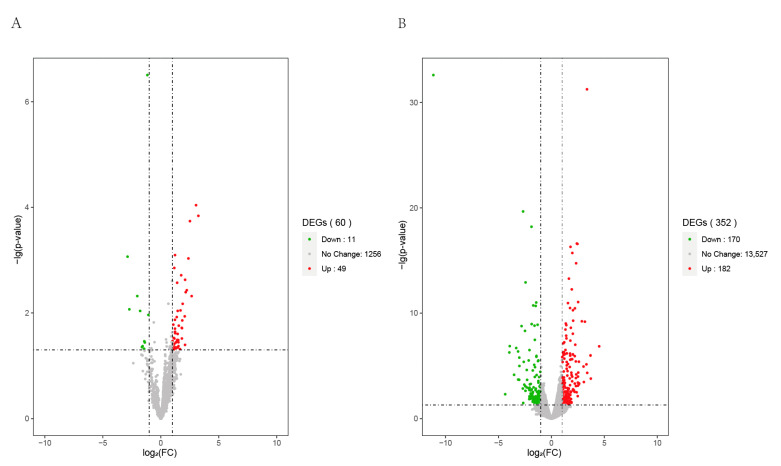
Volcanic map of differentially expressed lncRNAs and mRNAs in goat skin in different periods (Day 45 and Day 108): (**A**) Among the differentially expressed lncRNAs, 49 were up-regulated and 11 were down-regulated; (**B**) Among the differentially expressed mRNAs, 182 were up-regulated and 170 were down-regulated.

**Figure 5:**
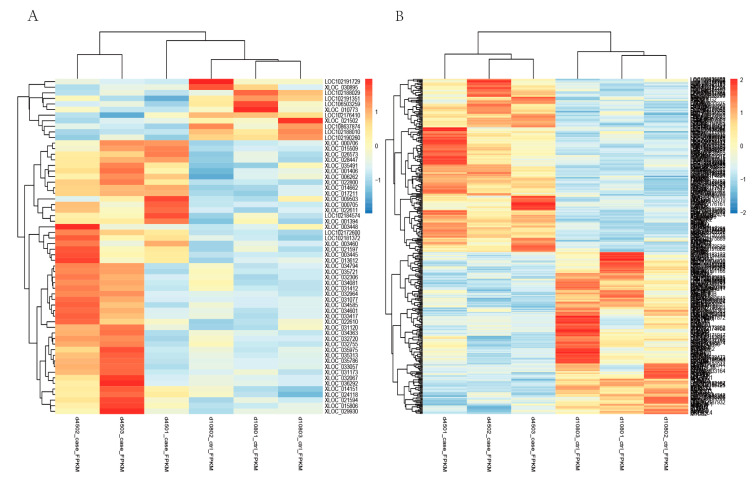
Cluster analysis of differentially expressed mRNAs and lncRNAs from the goat skin samples at different stages (day 45 and day 108): (**A**) the cluster analysis of FPKM values of 60 differentially expressed lncRNAs; (**B**) the cluster analysis of FPKM values of 352 differentially expressed mRNAs. Note: The color of the heat map represents the level of gene expression. The horizontal axis represents the clustering results of different samples and samples, and the vertical axis represents the gene name and the clustering results of genes.

To evaluate the expression level of the transcripts, the FPKM values of each sample transcript were normalized. We found that the expression of mRNAs was higher than that of the lncRNAs in the six goat skin samples. For the differentially expressed lncRNAs and mRNAs analysis, the screening threshold was set to a *p*-value < 0.05 and |log_2_FC| ≥ 1. In total, 182 up-regulated and 170 down-regulated mRNAs were identified; 49 up-regulated and 11 down-regulated lncRNAs were found from skins of 45-day-old (curving wool) and 108-day-old goats (slight-curving wool) (Figure 4). Hierarchical cluster analysis of the differentially expressed genes of the two groups was performed using the FPKM values of mRNAs and lncRNAs (Figure 5, Supplementary Tables S1 and S2). The results showed that the expression pattern of individuals in the same group were similar.

### 3.4. Differential lncRNAs Target Gene Prediction

To study the potential roles of lncRNAs in goat wool bending, the target genes of differentially expressed lncRNAs were predicted. In this study, 16 cis-acting target genes and 333 trans-acting target genes were predicted. Of which, 28 common genes were found in the significantly differential expressed mRNAs.

### 3.5. Weighted Gene Co-Expression Network Analysis (WGCNA)

**Figure 6:**
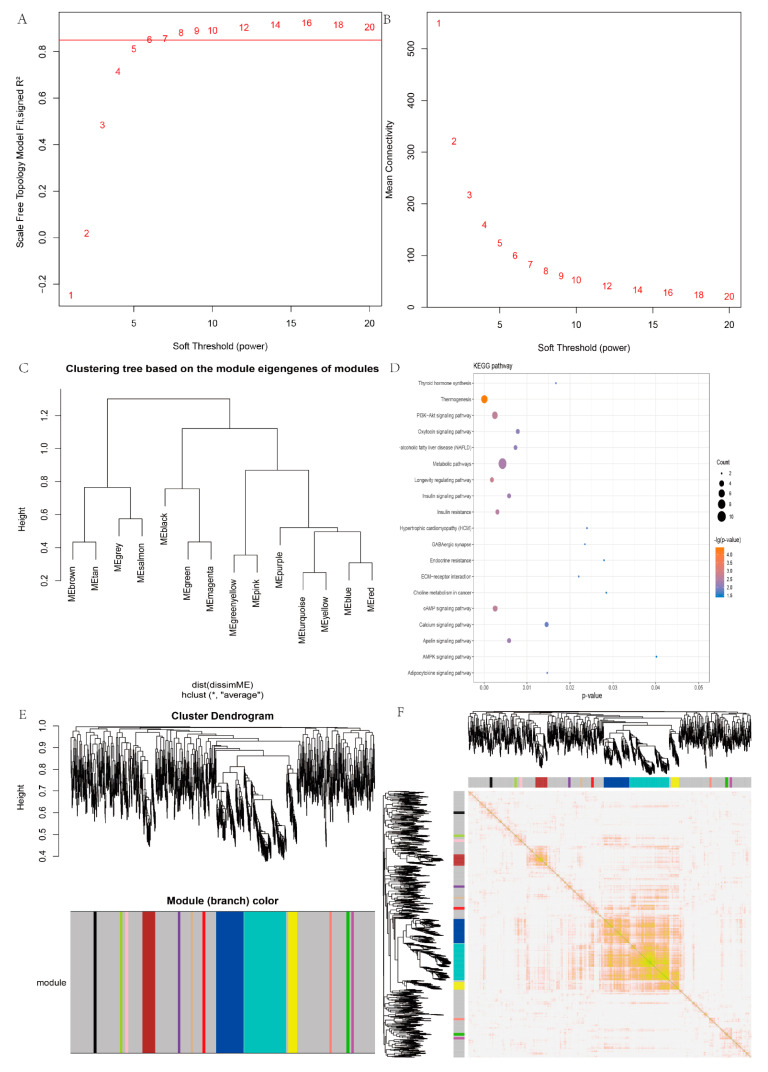
WGCNA of lncRNAs. (**A**,**B**) Calculating soft threshold; (**C**) clustering tree based on the module eigengenes of modules; (**D**) KEGG of the target gene of lncRNA in the turquoise module; (**E**) gene hierarchical clustering and module division; (**F**) correlation analysis of modules.

We compared the differences between the two groups of lncRNAs, and a total of 1316 lncRNAs were performed for WGCNA. The soft power β was selected as 16 to perform WGCNA, which ensured a scale independence > 0.85 (Figure 6A,B). Clustering based on the module eigengenes of MEyellow and MEturquoise were clustered together according to height (the height = 0.1) (Figure 6C). According to the clustering results, a total of 13 modules (yellow, blue, turquoise, tan, salmon, red, purple, pink, magenta, greenyellow, green, brown, and black) were identified, of which 183 differential lncRNAs were in the turquoise module (Figure 6D). Detailed information about the gene clustering module is shown in Supplementary Materials Table S8. Through the correlation test of the modules, it was found that there were significant correlations among the three modules of turquoise, blue, and yellow (Figure 6E). In addition, we carried out KEGG enrichment analysis of the target genes of differential expressed lncRNAs in the turquoise module. These genes were significantly enriched in several pathways related to follicle development, such as PI3K-Akt and MAPK signal pathway (Supplementary Table S9), suggesting the turquoise module might play vital roles in regulating wool bending. As the expression pattern of the genes in a module was similar, other genes in the turquoise module may participate in modulating goat wool bending. In addition, several genes associated with hair follicle development, such as *LOC102172600* and *LOC102191729* were found to cluster into the yellow module. Thus, we speculated the other genes in the yellow module may also be involved in the development of hair follicles and wool bending. The functions of these genes need to be further explored.

### 3.6. qRT-PCR Verification Result

**Figure 7:**
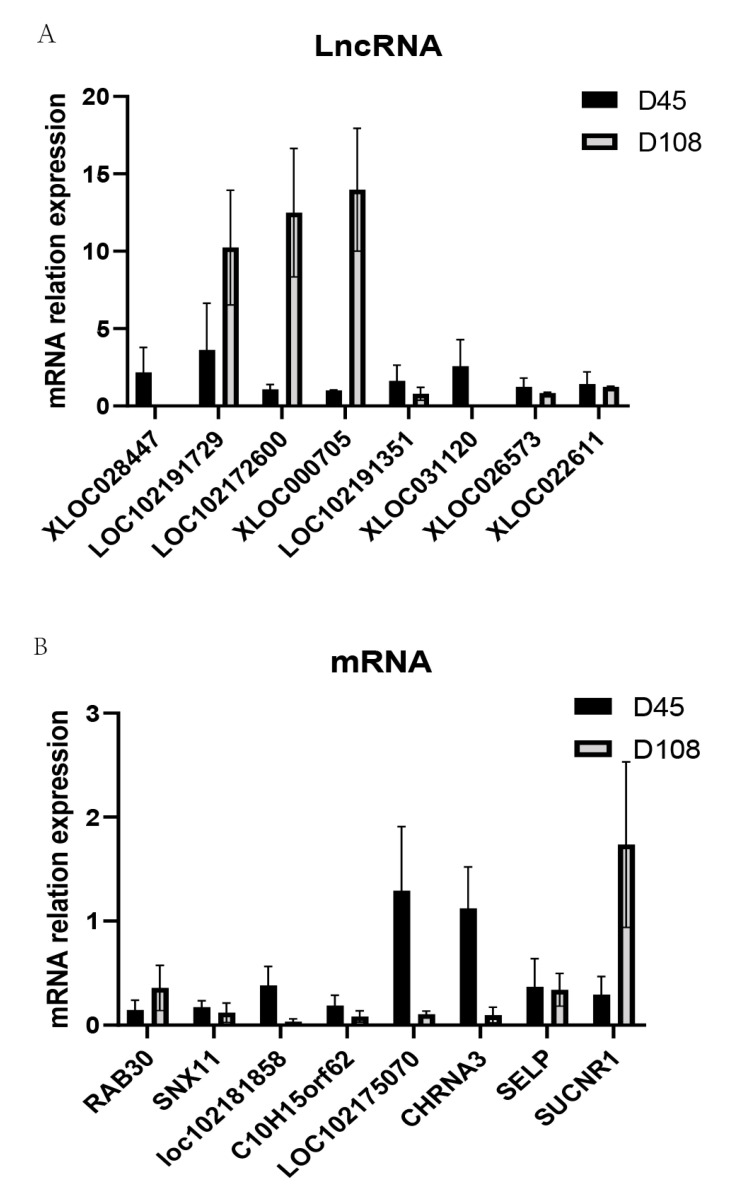
Verification of differentially expressed lncRNAs (**A**) and mRNAs (**B**) by qRT-PCR.

In order to validate the transcriptomic data and explore the regulatory relationship of lncRNAs and its target genes, the expression patterns of eight lncRNAs and predicted target genes at two stages were verified by real-time q-PCR. All the primers are listed in Table 2. According to the expression level and the function of the predicted target genes, eight lncRNAs and mRNAs were selected for q-PCR validation. For lncRNAs, the expression of *XLOC028447, XLOC031120, LOC102191351, XLOC026573* and *XLOC022611* was higher in the group of day 45 compared with the group of day 108; the *LOC102191729, LOC102172600* and *XLOC000705* expressions were lower in the group of day 45. Among the target genes regulated by DE lncRNAs, only the expression level of *RAB30* and *SUCNR1* increased and the expression level of the rest decreased gradually during the growth process. The results were consistent with RNA-seq data (Supplementary Figure S1), indicating our RNA-seq data were reliable (Figure 7).

### 3.7. GO and KEGG Enrichment Analysis of lncRNAs Targets

To further explore the lncRNAs potential function in goat wool bending changes at different developmental stages, we performed gene ontology (GO) enrichment analysis and Kyoto Encyclopedia of Genes and Genomes (KEGG) for the differentially expressed lncRNAs. Firstly, we predicted the target genes of cis- and trans-acting lncRNAs (Supplementary Table S3), which were subjected to GO and pathway analyses.

**Figure 8:**
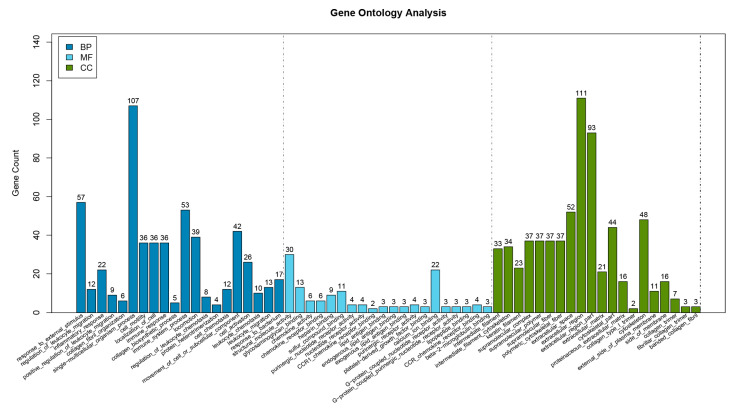
GO classification of differentially expressed lncRNAs target genes. Genes were enriched in cellular component (CC), molecular functional (MF), and biological function (BP).

**Figure 9:**
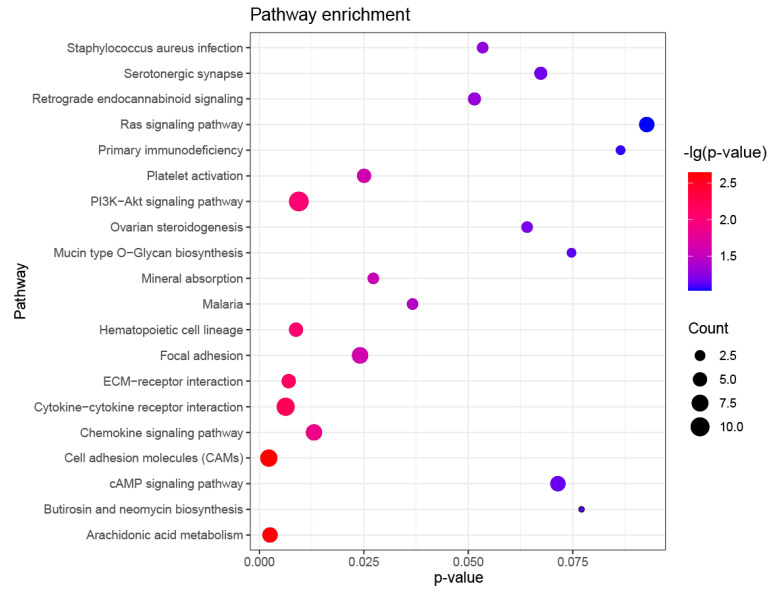
The top 20 pathways with the highest enrichment degree of up-regulated and down-regulated genes.

The GO analysis showed that the differential genes were significantly enriched in 298 GO terms. The top 20 GO Terms including cellular component (CC), molecular functional (MF), and biological function (BP) categories were displayed (Figure 8). Notably, the five most prominent GO terms were collagen fibril organization (GO:0030199), intermediate filament cytoskeleton (GO:0045111), keratin filament (GO:0045095), glycosaminoglycan binding (GO:0005539), and intermediate filament (GO:0005882), which were related to organic substance metabolic, regulation of stimulus, and cellular metabolic process, as well as hair follicle growth and development and hair formation. It indicated that these significantly enriched GO terms were associated with hair formation, growth, and development. KEGG pathway analysis demonstrated that 164 pathways were significantly enriched, such as TNF, MAPK, and PI3K-Akt, which play an important role in the regulation of hair follicle development (Figure 9). The above results suggest that DE lncRNAs and target genes may exert an important function in the wool bending trait of the Zhongwei goat.

## 4. Discussion

In recent years, increasing evidence has demonstrated that lncRNAs play important roles in the development of various tissues, such as the brain [29], liver [30], heart [31], testis [32], ovary [33], kidney, uterus [34], muscle [35], mammary gland [36], and inner ear [37]. However, few studies have been conducted on the potential role of lncRNAs in mammalian hair follicle development and wool curvature. The regulation mechanism underlying the wool curvature of the Zhongwei goat changes with growth is more complex. In this study, to explore the potential effect of lncRNAs on the changes of hair phenotype in goats at different growth stages, the expression profiles of the lncRNAs of goat skin at day 45 and day 108 were constructed. Based on the sequencing data, we found that the length of lncRNAs was mainly between 200–1000 bp; the of lncRNAs included 2–4 exons, while mRNAs consisted of 0–20 exons; the ORF length of lncRNAs is mostly between 100–1000 bp and the ORF length of mRNAs is between 100–2000 bp; and the expression level of lncRNAs related to hair follicles is lower than that of protein-coding genes. These results are consistent with the basic characteristics of previous studies on lncRNAs, further illustrating the reliability of our data and the accuracy of identifying lncRNAs. Combining the differentially expressed mRNAs with lncRNAs and its target genes, the most significant GO terms and KEGG pathway analysis indicated that 164 pathways were enriched, such as TNF, MAPK, and PI3K-Akt, which play an important role in the regulation of hair follicle development. From the results of qRT-PCR, the expression of lncRNAs and the target gene changed significantly in different growth stages. The above results suggest DE lncRNAs and target genes might exert an important function on the wool bending of Zhongwei goats.

Hair follicles, acting as the basic organ of hair growth, are regulated by numerous signal pathways during the process of growth and development, which determine wool phenotypic characteristics. Previous studies have found that signal pathways such as PI3K-Akt [38], Chemokine [39], cAMP [40], and Ras [41] play direct or indirect roles in the development of hair follicles. In our enrichment analysis of 60 differential lncRNAs target genes and 352 mRNAs, PI3K-Akt, Chemokine, cAMP, and Ras pathways were among the top 20 pathways enriched. LncRNAs exert the function through interacting with DNA, RNA, and protein at multiple levels of epigenetic, transcriptional, and post-transcriptional levels [8]. Several lncRNAs relevant to skin biology have been reported, such as Terminal differentiation–induced ncRNA (TINCR), Anti-differentiation ncRNA (ANCR), and lncRNA H19 (a 2322 bp nuclear intergenic lncRNAs) [42]. To validate and explore the potential regulation relationship of lncRNAs and its target gene, eight different lncRNAs and mRNAs were determined using the qPCR method. As expected, it was found that the expression of lncRNAs and the corresponding predicted target genes changed dramatically in different periods. We found that the expression of *LOC102172600* significantly increased with age, and the expression of its target gene *LOC102181858* decreased. In our study, the RNA-seq result showed *LOC102172600* and target gene *PRDM1* were significantly differential expressed at two stages. *PRDM1*, a core transcriptional regulator, regulates dermal papilla development to affect morphogenesis and postnatal hair cycle thorough the activation of dermal Wnt/β-catenin during hair follicle (HF) development [43]. *GORAB,* predicted target gene of *LOC102191729*, is necessary for hedgehog (HH) signaling pathway transduction in the process of hair follicle embryo morphogenesis [44]. Therefore, we infer that both *LOC102172600* and *LOC102191729* may have an important effect to regulate the goat wool curvature. The high expression of *SUCNR1,* a predicted gene of *LOC102176410*, may assist in regulating cell metabolism. We speculate that the gene *SUCNR1* may indirectly regulate the development of hair follicles, and it can be activated by succinic acid to affect the local pressure of cell metabolism [45]. These genes that indirectly regulate the degree of hair bending are also regulated by lncRNAs.

Previous studies showed that lncRNA5322 promotes the proliferation and differentiation of hair follicle stem cells by targeting the miR-21-mediated PI3K-Akt signal pathway [46], whereas the PI3K-Akt signal pathway has been found to regulate the development of hair follicles [39]. In our study, it was found that *F2R* and *FGF21,* the two differential genes, were significantly enriched in the PI3K-Akt signaling pathway. It is reported the upregulation of thrombin receptor *F2R* is related to the changes in the characteristics of the hair papilla cells, which disturb the plasticity of hair follicle dermal cells, thus regulating the function of hair follicle dermal cells [47]. Fibroblast growth factor 21 (*FGF21*) gene, which belongs to the *FGF* gene family that plays a role in hair development [48]. Some studies have shown that the fibroblast growth factor family is active in mouse skin at different growth stages, and changes dynamically in different patterns during the hair growth cycle [49,50]. The results show that differentially expressed mRNAs exert a certain role in regulating the wool bending changes during the growth of goats.

In the present study, we systematically identified the lncRNAs and mRNAs involved in goat (*Capra hircus*) hair bending across two postnatal stages based on the Ribo-Zero RNA-sequencing method. We subsequently characterized putative lncRNAs to elucidate their diverse features in order to provide an insight into the uncharacterized goat genome regions and their relationship with goat hair follicle development. To the best of our knowledge, we identified the expressed lncRNAs and mRNAs in different goat hair follicle developmental stages.

## 5. Conclusions

In conclusion, the lncRNAs profiling of skin from Zhongwei goats with different wool-bending phenotypes were constructed. Here, 60 differential lncRNAs and 352 differential mRNAs were identified between curving wool and slight-curving wool goats. By analyzing the biological functions of differential lncRNAs and mRNAs, a set of signal pathways and lncRNAs were identified to be involved in the development of hair follicles and wool curvature. Our finding can provide a basis for the study of the development of hair follicles and the mechanism of wool bending in Zhongwei goats.

## Figures and Tables

**Table 1:** Data quality control results of each sample.

Sample	Clean Bases	Clean Reads	Clean GC	Clean Q20 (%)	Clean Q30 (%)
d10801	14,610,168,300	48,700,561	46.05	99.01; 95.36	96.91; 90.02
d10802	18,156,167,100	60,520,557	45.11	99.03; 95.81	97.12; 91.05
d10803	19,196,462,400	63,988,208	46.45	99.02; 95.71	97.09; 90.82
d4501	16,044,002,400	53,480,008	47.09	99.03; 95.62	96.96; 90.53
d4502	15,798,806,400	52,662,688	46.22	98.96; 95.70	96.81; 90.81
d4503	15,817,844,400	52,726,148	45.44	98.97; 96.10	96.85; 91.52

**Table 2:** Sequence information of primers.

Type	Gene ID/Symbol	Forward Primer (5′-3′)	Reverse Primer (5′-3′)
mRNA	SUCNR1	GAGGAACAGGCAGCTCACTA	CCGGACATTTCGCATGACAT
	SNX11	AGAACCAAGAGCAGGAGACC	AAACCCGCATTTCTCTGGAG
	LOC102181858	CCCAATGGTCATCTTCTGCC	CCAGGGTTTTAGAGTGCCAT
	RAB30	GAATCCTTCCGTTGCCTTCC	GACCTCTCTCCTTTCAGCCA
	LOC102175070	TGTAGAAACCTGGTCCCTGT	CAGGCCTCTTTTGTCCGTTA
	CHRNA3	CGACTATGATGGGGCTGAGT	CTTTGATGATGGCCCACTCG
	SELP	CCGGCAAGTGGAATGATGAG	ACAGGAGCAGGTGTAGTTCC
	C10H15orf62	CAGACACCCTCCACCAATCT	CCCAGGTCCACTTTGAAGGA
lncRNA	LOC102191729	CATGATGAAGGGAGCACTGC	CATCAAGTCCCGCCTCATTG
	XLOC_028447	CGACAGAGCATGCATGTGAA	GCAATGACCGTGAGCCTTAG
	XLOC_031120	GGTTTCCCGCCTTTTCACAT	TGGGACTGCTGTAGGGAAAG
	LOC102172600	CTGCGTTGTCTCATCACTCC	GTTGTTCTTCGGAGGGCTTG
	LOC102191351	ACAGAGAAGAGCGATGTTGC	CTTCTAACCACTGGACTGCC
	XLOC_000705	GGAAACTACTATGGCGGCCT	AGCCACTTCCACAGAGAGAG
	XLOC_026573	ATCTTAGCCACTGGACCACC	ACCTGCTGAACCTGCTGTAT
	XLOC_022611	GTTGGCGAGTGTTCTGAGC	GAAGGCACCTCTCTCGATGA
Reference gene [28]	GAPDH	TGGCAAAGTGGACATCGTTG	GGACTCCACCACGTACTCAG

## Data Availability

The data of this article has been uploaded the data to the GSA database, and the data are publicly available. Database URL: https://ngdc.cncb.ac.cn, accessed on 18 September 2021. GSA accession: CRA005362.

## Supplementary Materials

The following are available online at https://www.mdpi.com/article/10.3390/ani11113326/s1. Table S1: List of differentially expressed lncRNAs of samples in two groups. Table S2: List of differentially expressed mRNAs of samples in two groups. Table S3: List of target genes for cis- and trans- actions of differential lncRNAs. Table S4: List of GO enrichment analysis of differentially expressed mRNAs. Table S5: List of KEGG enrichment analysis of differentially expressed mRNAs. Table S6: List of GO enrichment analysis of target genes of differentially expressed lncRNAs. Table S7: List of KEGG enrichment analysis of target genes of differentially expressed lncRNAs. Table S8: List of gene clustering module. Table S9: List of the KEGG of the target gene of lncRNA in the turquoise module. Figure S1: Sequencing results Normalized lncRNAs and mRNAs expression.
