# Supplementary figures and images for: Integrated Analysis of lncRNA and mRNA Reveals Novel Insights into Wool Bending in Zhongwei Goat
Source: Animals (Basel). 2021 Nov 22;11(11):3326. doi: 10.3390/ani11113326 (PMC8614501; doi:10.3390/ani11113326)

Supplementary Materials:

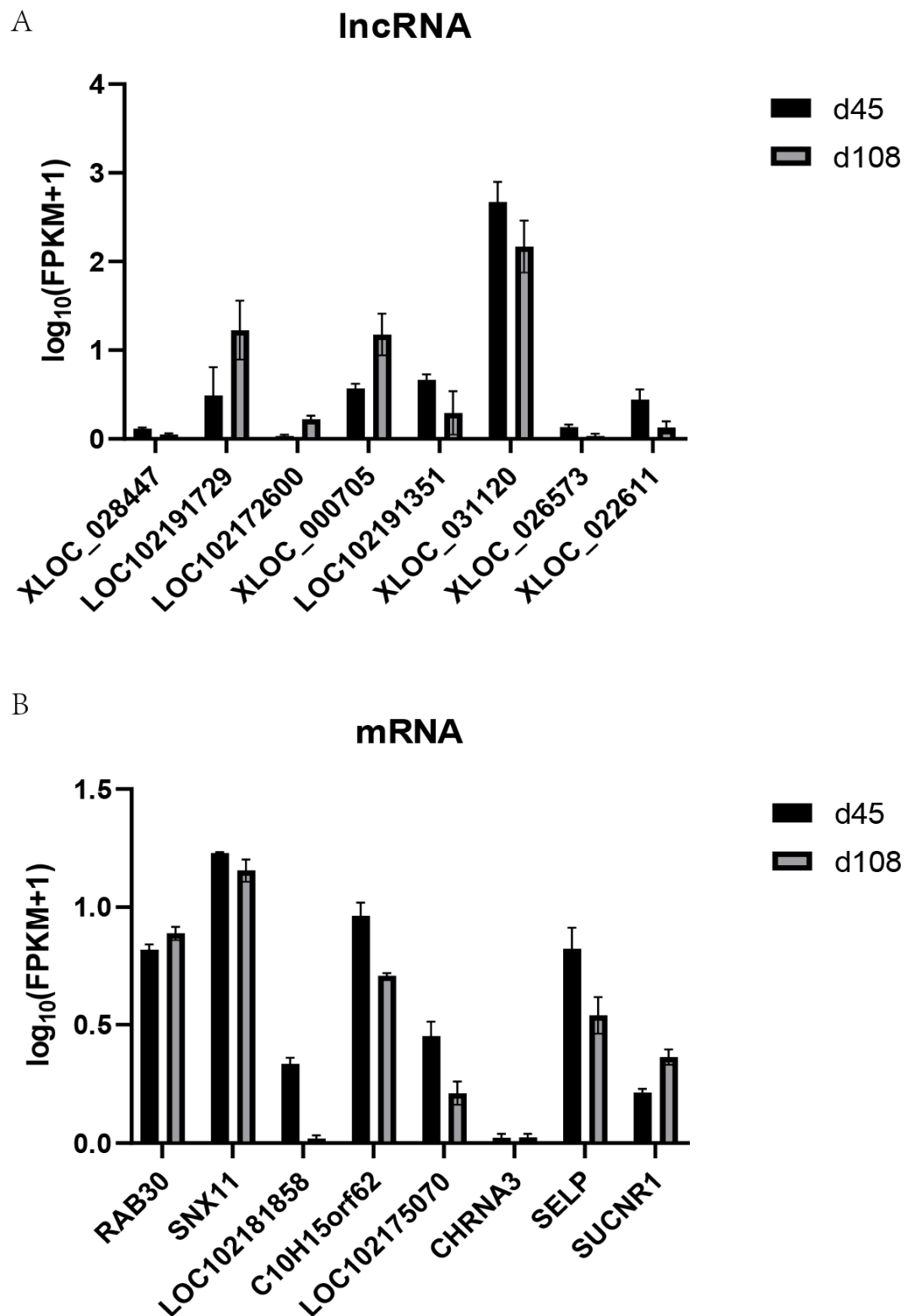

Figure S1: Sequencing results Normalized lncRNA and mRNA expression

Supplement: Supplementary file 1 [file animals-11-03326-s001.zip › Figure S1 Sequencing results Normalized lncRNA and mRNA expression.pdf]
